# Supplementary material for: Prothrombinase processivity is conferred by substrate allostery
Source: EMBO J. 2026 Apr 22;45(11):3954–77. doi: 10.1038/s44318-026-00782-4 (PMC13226733; doi:10.1038/s44318-026-00782-4)
Supplement: Supplementary file 1 — Appendix [file 44318_2026_782_MOESM1_ESM.pdf]

Appendix for

Prothrombinase processivity is conferred by substrate allostery

Table of Contents

Item		
Appendix Figure S1	AlphaFold 3 results for prethrombin-2 and the a2-peptide	1
Appendix Figure S2	Comparison of prothrombinase structures	2
Appendix Figure S3	Tilt of prothrombinase in the free and substrate-bound states	3
Appendix Figure S4	Rotation of meizothrombin requires release of F1	4
Appendix Figure S5	FSC plots	5
Appendix Table S1	Factor Xa (M17) light chain contacts with fVa	6
Appendix Table S2	Factor Xa (M17) SP domain interactions with fVa	7-9
Appendix Table S3	Prothrombin interactions with fVa	10-11
Appendix Table S4	M17 SP domain interactions with prothrombin	11-12
Appendix Table S5	Prothrombin interactions with fXa	12-13
Appendix Table S6	Meizothrombin light chain (P) interactions with fVa	13
Appendix Table S7	Meizothrombin light chain (P) interactions with fXa	13-15
Appendix Table S8	Meizothrombin SP domain (Q) interactions with fXa	15
Appendix Table S9	F1 interactions with the SP in prothrombin	15-16
Appendix Table S10	F1 clashes (<2.3Å) with the SP in meizothrombin	16
Appendix Table S11	K2 contacts (<5Å) with the SP in prothrombin	16-17
Appendix Table S12	K2 contacts (<5Å) with the SP in meizothrombin	17-18
Appendix Table S13	K2 in prothrombin position clashes with meizothrombin	19
Appendix Table S14	Primers	19
Appendix Table S15	Assembly of pET23- M17 (EGF2-SP) S195A construct	19

Appendix Figure S1: AlphaFold 3 results for prethrombin-2 and the a2-peptide. Screenshot of the AlphaFold 3 results using the sequences of prethrombin-2 and a peptide corresponding to the C-terminal acidic region of the a2-loop. The result is colored according to the confidence score (key above), and the 2D relative positional confidence plot on the right.

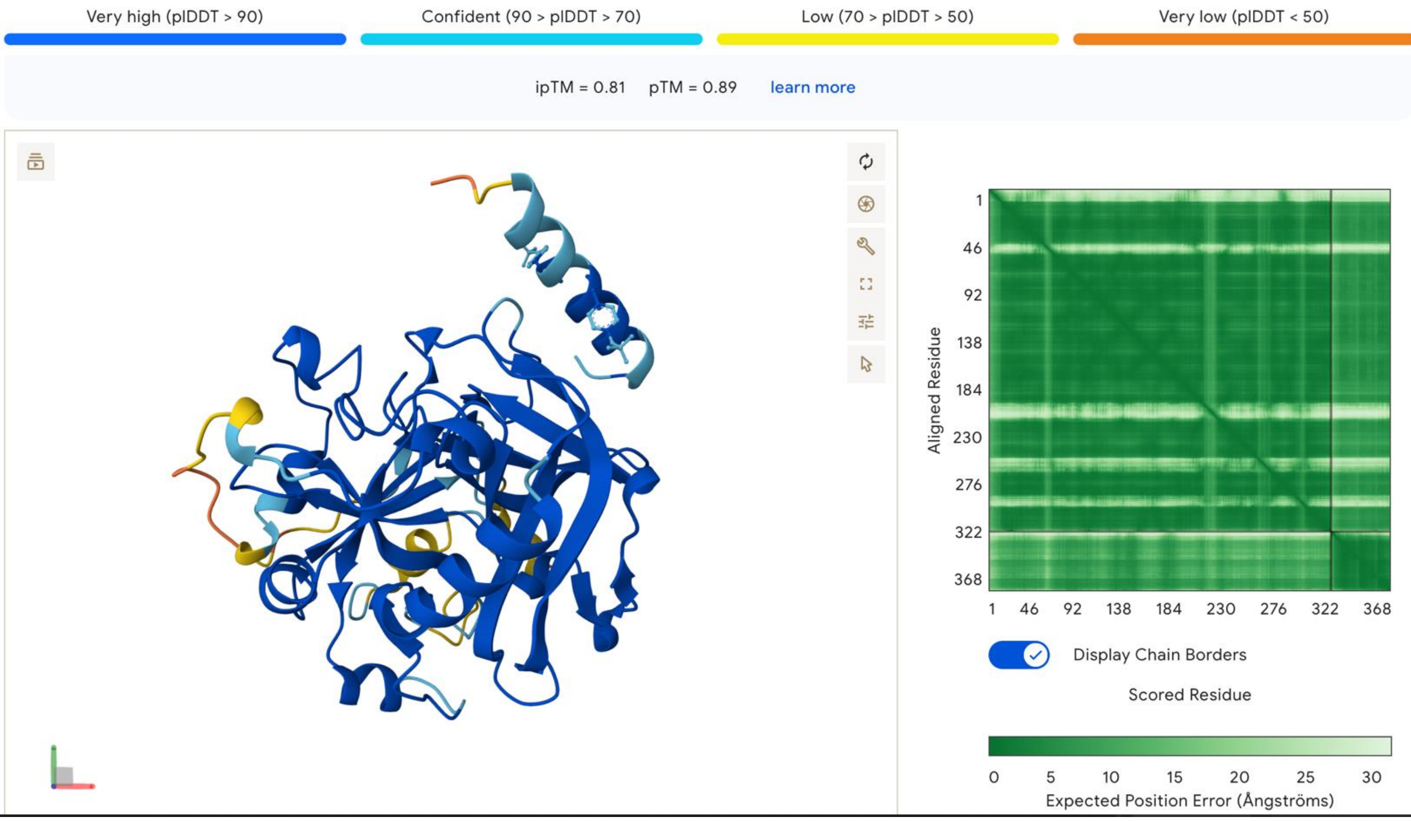

Appendix Figure S2: Comparison of prothrombinase structures. Stereo view of the superposition of the structures of prothrombinase alone (9I2H; gray with Gla-EGF1 removed), from the prothrombin complex (9TLE; yellow) and from the meizothrombin complex (9TLG; orange).

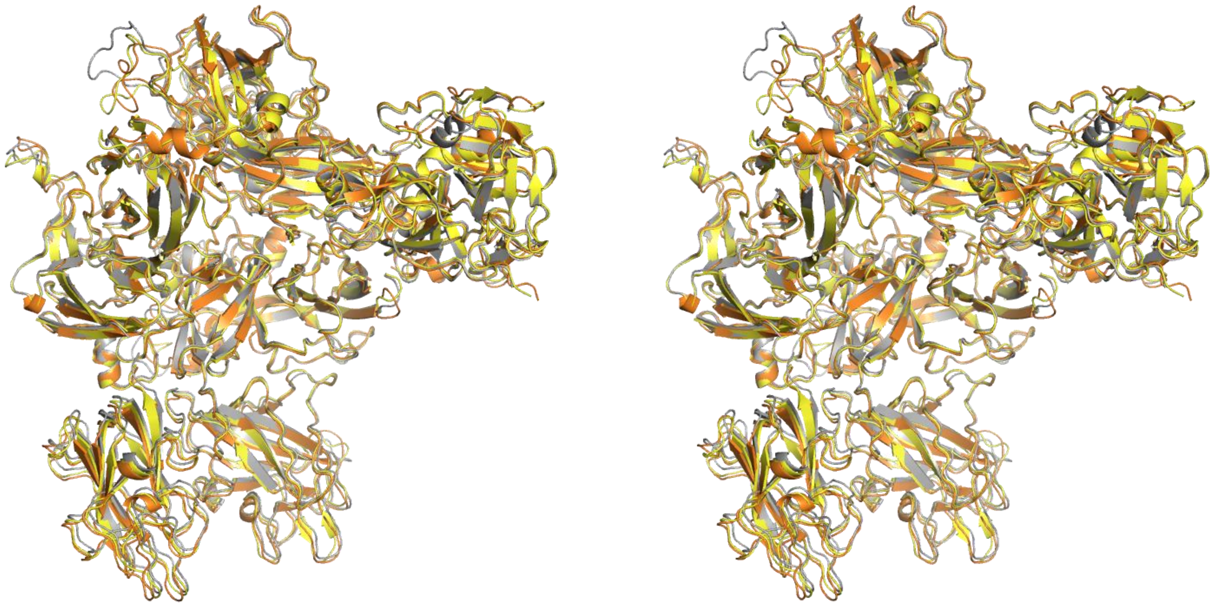

Appendix Figure S3: Tilt of prothrombinase in the free and substrate-bound states. A side view of the full-length prothrombinase structure (Gla and EGF1 as in 9I2H) and a model of a PL bilayer illustrates the potential range of movement. The near perpendicular orientation of substrate-free prothrombinase is shown on the left and the tilting required to accommodate PL-bound prothrombin is on the right. Roughly 20 degrees forward tilt is required.

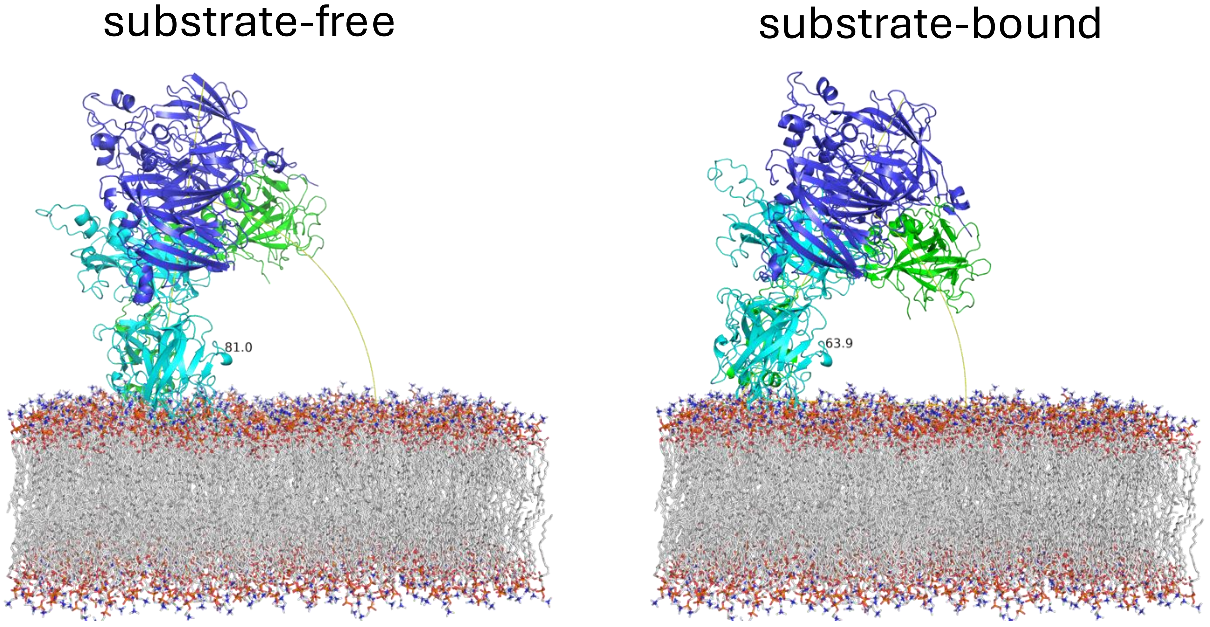

Appendix Figure S4: Rotation of meizothrombin requires release of F1. The prothrombin-to-meizothrombin transition modelled on a PL bilayer surface illustrates the movement of the F1 region (Gla and K1) if dissociation from the SP domain did not occur. Prothrombin is colored as in other figures (fVa heavy chain in blue and light chain in cyan; fXa in green; prothrombin in yellow and meizothrombin without change in F1-SP interaction in orange.

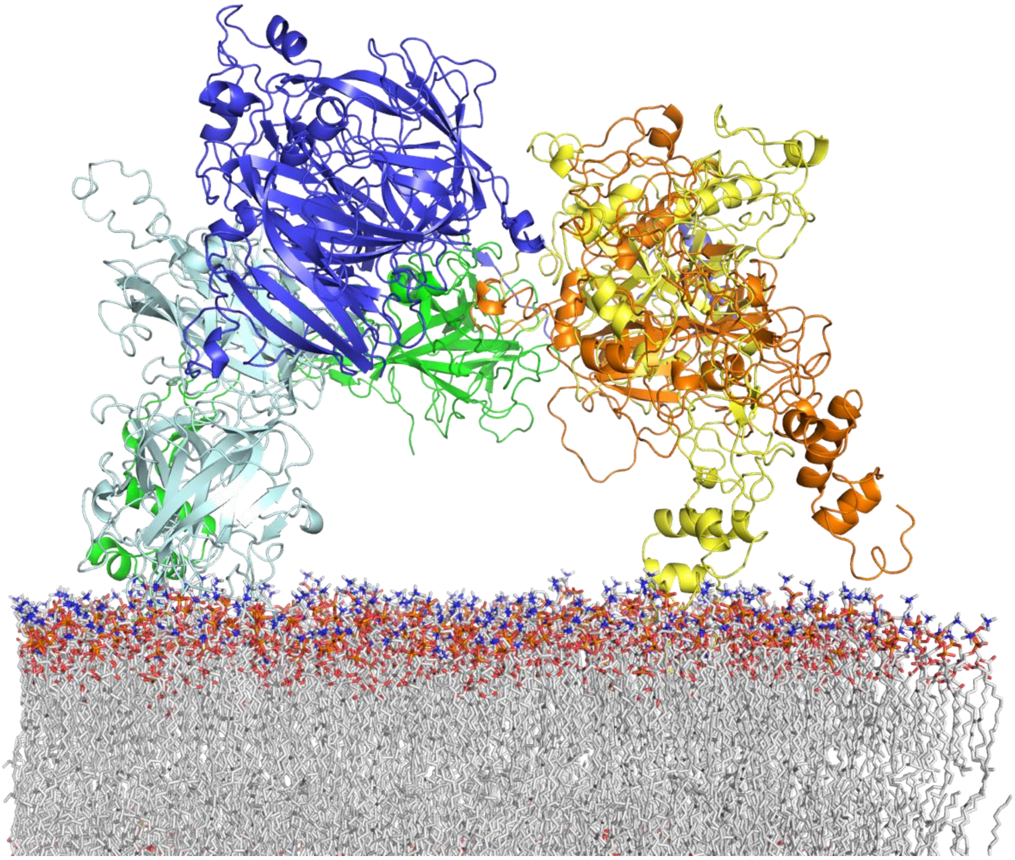

Appendix Figure S5: FSC plots. Fourier Shell Correlation (FSC) plots for the maps of the prothrombinase-prothrombin (A) and prothrombinase-meizothrombin (B) complexes.

A

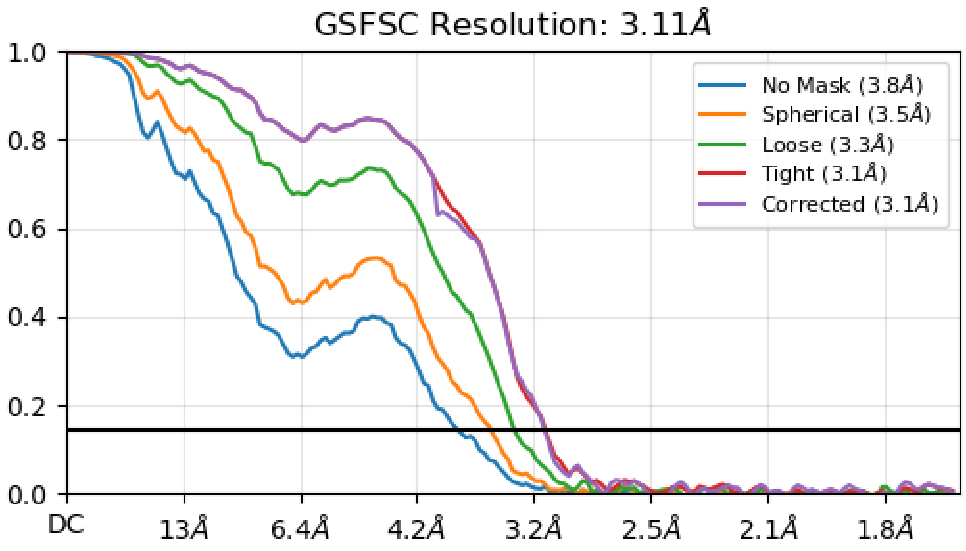

B

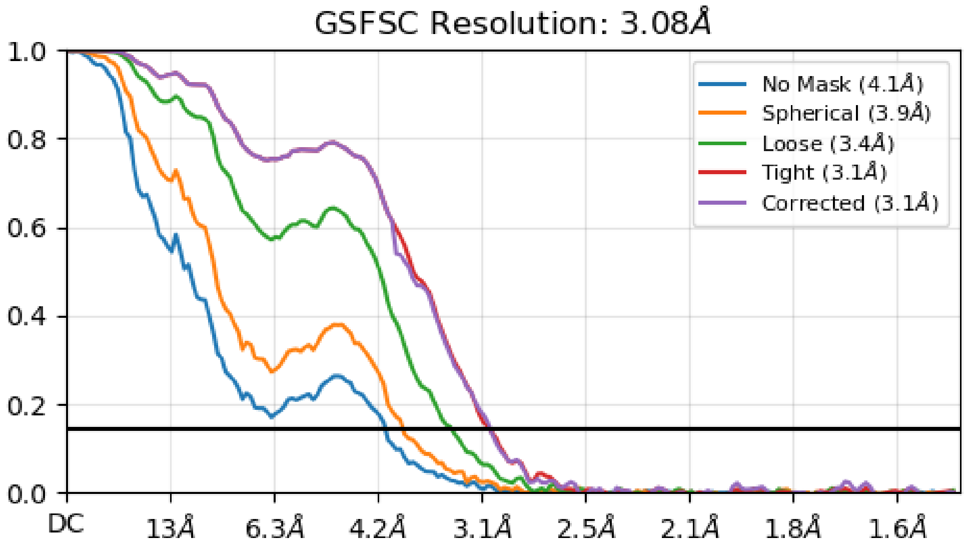

Appendix Table S1: Factor Xa (M17) light chain contacts with fVa. Contacting residues identified by the program ‘contact’ are listed. Interaction type as identified by PISA are in the last column with HB for hydrogen bond and SB for salt-bridge.

fXa		fVa		
Domain	Residue	Domain	Residue	Interaction Type
EGF2	Met85	A3	Gln1629	
			Thr1679	
			Val1681	
	Arg86	A3	Ser1652	HB
			Ser1653	
			Trp1665	
			Glu1668	SB
			Tyr1678	HB
			Thr1679	HB
	Leu88	A3	Glu1650	
			Ser1652	
			Phe1666	
			Val1681	
			His1683	
	**Ser90Arg**	A2	Asn629	2HB
		A3	Ser1648	HB
			Tyr1649	
			Glu1650	
			His1683	
	**Leu91Ala**	A3	Val1627	
			Arg1551	
			His1683	
	**Asp92Phe**	A3	Asn1547	
			Gly1549	
			Asn1550	
			Arg1551	
			Val1627	
	**Glu102Arg**	A3	Glu1650	4SB
			Ser1652	
			Phe1666	
	**Glu103Val**	A3	Trp1665	
	Gln104	A3	Trp1665	
	**Asn105Ser**	A3	Trp1665	

Appendix Table S2: Factor Xa (M17) SP domain interactions with fVa. Contacting residues identified by the program ‘contact’ are listed. Interaction type as identified by PISA are in the last column with HB for hydrogen bond and SB for salt-bridge. Chymotrypsin numbering used for SP, and M17 mutations are in bold.

fXa	fVa		
Chymo #ing	Domain	Residue	Interaction Type
Glu36	a2-loop	Ser673	
		Thr674	
		Val675	
Tyr60	a2-loop	Phe668	
Gln61	a2-loop	Phe668	
		Glu669	
		Pro670	
Ala61a	a2-loop	Ser673	
Lys62	a2-loop	Glu669	
		Pro670	
		Pro671	HB
		Glu672	SB
		Ser673	
		Thr674	
Phe64	a2-loop	Pro670	
Lys65	a2-loop	Thr674	
Glu84	a2-loop	Thr674	
Val85	a2-loop	Pro670	
		Pro671	
Glu86	a2-loop	Glu669	
		Pro670	
		Pro671	
Val87	a2-loop	Ile667	
		Phe668	
		Pro670	
Val88	a2-loop	Glu666	
		Ile667	
		Phe668	2HB
		Glu669	
		Pro670	
Ile89	a2-loop	Tys665	
		Glu666	
		Ile667	
Lys90	a2-loop	Tys665	
		Glu666	2HB
		Phe668	
His91	a2-loop	Ser664	
		Tys665	
		Glu666	
Asn92	a2-loop	Ser664	
		Tys665	
		Glu666	
Lys109	a2-loop	Pro671	
Arg125	A2	Lys655	
		Cys656	HB
	a2-loop	Ile657	
		Pro658	

		Asp659	
Asp126	A2	Lys655	
**Glu129Asn**	A2	Phe576	
		Asp577	
		Lys655	
**Ser130Glu**	A2	Phe576	
		Val630	
		Lys655	
Met131b	A2	Asp577	
**Thr132Lys**	A2	Phe576	
		Asp577	SB
		Asp628	2SB
	A3	Ser1546	
Gln133	A3	Ser1546	
Asp134	A3	Ser1546	
Asp164	A3	Glu1686	
Arg165	A2	Asp513	3SB
		Asp577	
		Thr579	
**Asn166His**	A2	Gln509	
		Ala511	HB
		Ala512	
		Thr579	
		Gln581	
		Thr624	
**Lys169Met**	A2	Arg510	HB
		Ala511	
		Ala512	
		Asp513	
		Thr579	
Leu170	A2	Gln471	
		Arg510	HB
		Ala511	
Ser171	A2	Arg510	
Ser172	A2	Arg510	HB
**Ser173Asp**	A2	Arg501	5SB
		Arg510	
Phe174	A2	Arg510	HB
**Ile175Arg**	A2	Asp504	3SB
		Ile508	
		Gln509	HB
		Arg510	
Ile176	A2	Arg510	
		Asp513	
Gln178	A2	Asp513	HB
		Asp578	
Thr232	a2-loop	Asp659	
**Ala233Arg**	a2-loop	Asp659	5SB
		Glu662	
Phe234	a2-loop	Asp659	
		Glu662	
Leu235	a2-loop	Glu662	
Lys236	a2-loop	Pro658	HB

		Asp660	HB
		Glu662	
Trp237	a2-loop	Glu662	
		Asp663	
		Ser664	
		Tys665	
Arg240	a2-loop	Glu662	
		Asp663	
		Tys665	HB
Ser241	a2-loop	Tys665	HB
Lys243	a2-loop	Tys665	
Thr244	a2-loop	Tys665	
		Ile667	
Gly246	a2-loop	Ile667	

Appendix Table S3: Prothrombin interactions with fVa. Contacting residues identified by the program 'contact' are listed. Interaction type as identified by PISA are in the last column with HB for hydrogen bond and SB for salt-bridge. Chymotrypsin numbering is given for residues in the SP domain.

Prothrombin		fVa		
Mature #ing	Chymo #ing	Domain	Residue	Interaction Type
Glu253	-	a1-loop	Arg313	
Glu254	-	a1-loop	Arg313	
Glu255	-	a1-loop	Arg313	
Thr256	-	a1-loop	Arg313	
Gly257	-	a1-loop	Arg313	
Leu260	-	a1-loop	Arg316	
Asp261	-	a1-loop	Arg316	
		a1-loop	Arg317	
Arg266	-	A2	Arg321	
Lys341	36	a2-loop	Leu706	
Gln344	38	a2-loop	Leu706	HB
			Gly707	HB
			Ile708	
			Arg709	
Leu380	65	a2-loop	Leu706	
			Ile708	
Arg382	67	a2-loop	Ile708	
			Arg709	SB (C-term)
Lys385	70	a2-loop	Arg709	
Arg388		a2-loop	Arg709	
Thr389	74	a2-loop	Arg709	
Arg390	75	a2-loop	Arg709	
Tyr391	76	a2-loop	Gln699	
			Asn700	HB
			Ala703	
			Ile708	
			Arg709	HB
Arg393	77a	a2-loop	His682	2HB
			Asp683	
			Arg684	
			Leu685	
			Tys696	HB
			Asn700	
Asn394	78	a2-loop	Leu685	
			Glu686	
			Tys696	
			Asn700	
Glu396	80	a2-loop	Gln699	
Lys397	81	a2-loop	Asp695	
			Gln699	
Ile398	82	a2-loop	Tys698	
			Gln699	
			Leu702	
			Ile708	
Ser399	83	a2-loop	Tys698	
Met400	84	a2-loop	Tys698	
			Leu702	

Lys427	110	a2-loop	Asp695	
			Tys698	HB
Gln451	131	a1-loop	Arg313	HB
			Arg317	
Ala452	132	a1-loop	Arg317	
Glu489	164	a1-loop	Arg313	

Appendix Table S4: M17 SP domain interactions with prothrombin. Contacting residues identified by the program 'contact' are listed. No HB or SB identified by PISA. Chymotrypsin numbering used for SP domain of fXa. Bold if not P4-P2′ (317-322). Note only 5 other residues contact fXa, so only 11 residues in total on prothrombin.

fXa	Prothrombin	
Chymo #ing	Residue	Interaction Type
Phe41	Ile321	
	Val322	
Cys42	Arg320	
	Ile321	
His57	Gly319	
	Arg320	
	Ile321	
Cys58	Ile321	
Tyr60	**Lys307**	
Lys96	**Arg310**	
	Ile317	
Glu97	**Arg310**	
	Ile317	
Tyr99	Ile317	
	Gly319	
	Ile321	
Arg143	Val322	
Lys148	**Tyr510**	
Gln151	Val322	
Phe174	**Ser315**	
	**Tyr316**	
	Ile317	
Asp189	Arg320	
Ala190	Arg320	
Cys191	Arg320	
Gln192	Asp318	
	Gly319	
	Arg320	
	Ile321	
	Val322	
Gly193	Arg320	
	Ile321	
	Val322	
Asp194	Arg320	
Ala195	Arg320	
	Ile321	
Val213	Arg320	
Ser214	Arg320	

Trp215	Ile317	
	Asp318	
	Gly319	
	Arg320	
Gly216	Asp318	
	Gly319	
	Arg320	
Glu217	Asp318	
	Arg320	
Gly218	Asp318	
	Arg320	
Arg222	**Tyr316**	
Tyr225	Arg320	
Gly226	Arg320	
Ile227	Arg320	
Tyr228	Arg320	

Appendix Table S5: Prothrombin interactions with fXa. Contacting residues identified by the program 'contact' are listed. No HB or SB identified by PISA. Chymotrypsin numbering used for SP domain of fXa.

Prothrombin	fXa	
Residue	Chymo #ing	Interaction Type
Lys307	Tyr60	
Arg310	Lys96	
	Glu97	
Ser315	Phe176	
Tyr316	Phe176	
	Arg222	
Ile317	Lys96	
	Glu97	
	Tyr99	
	Phe174	
	Trp215	
Asp318	Gln192	
	Trp215	
	Gly216	
	Glu217	
	Gly218	
Gly319	His57	
	Tyr99	
	Gln192	
	Trp215	
	Gly216	
Arg320	Cys42	
	His57	
	Asp189	
	Ala190	
	Cys191	
	Gln192	
	Gly193	
	Asp194	
	Ala195	

	Val213	
	Ser214	
	Trp215	
	Gly216	
	Glu217	
	Gly218	
	Tyr225	
	Gly226	
	Ile227	
	Tyr228	
Ile321	Phe41	
	Cys42	
	His57	
	Cys58	
	Tyr99	
	Gln192	
	Gly193	
	Ala195	
Val322	Phe41	
	Arg143	
	Gln151	
	Gln192	
	Gly193	
Tyr510	Lys148	

Appendix Table S6: Meizothrombin light chain (P) interactions with fVa. Contacting residues identified by the program 'contact' are listed. 0 HB and 2 SB identified by PISA. Chymotrypsin numbering used for SP domain of fXa.

Meizothrombin	fVa	
Residue	Residue	Interaction Type
Asp261	Glu314	
Ser264	Arg317	
Asp265	Arg317	2SB
Arg266	His318	

Appendix Table S7: Meizothrombin light chain (P) interactions with fXa. Contacting residues identified by the program 'contact' are listed. 13 HB and 3SB identified by PISA. Chymotrypsin numbering used for SP domain of fXa.

Meizothrombin	fXa	
Residue	Chymo #ing	Interaction Type
Arg266	Asp173	SB
	Phe174	
Ala267	Phe174	
Ile268	Lys96	
	Glu97	
	Thr98	
	Tyr99	
	Phe174	
	Trp215	

Glu269	Tyr99	
	Gln192	HB
	Trp215	
	Gly216	HB
	Glu217	
	Gly218	HB
Gly270	His57	
	Tyr99	
	Gln192	HB
	Ser214	
	Trp215	
	Gly216	
Arg271	Phe41	
	Cys42	
	His57	
	Asp189	3SB
	Ala190	HB
	Cys191	
	Gln192	
	Gly193	HB
	Asp194	HB
	Ala195	HB
	Val213	
	Ser214	
	Trp215	
	Gly216	
	Glu217	
	Gly218	HB
	Cys220	
	Gly226	
	Ile227	
Thr272	Phe41	HB
	His42	
	His57	
	Cys58	
	Gln61	HB
	Arg143	
	Gln151	
	Gln192	
	Gly193	
	Ala195	
Ala273	Phe41	HB
	Gln61	HB
	Gln151	
	Gly193	
Thr274	Gln61	
Ser275	Gln61	
Ser303	Glu37	
Arg310	Arg150	
Leu313	Lys148	
	Arg150	
Glu314	Arg150	
Tyr316	Lys148	

Ile317	His145	
	Glu147	
	Lys148	
	Gly149	
Asp318	His145	
Arg320	Arg150	

Appendix Table S8: Meizothrombin SP domain (Q) interactions with fXa. Contacting residues identified by the program 'contact' are listed. 3 HB and 0 SB identified by PISA. Chymotrypsin numbering used for SP domain of fXa.

Meizothrombin		fXa	
Residue	Chymo #ing	Chymo #ing	Interaction Type
448	Ser129b	Lys148	HB
449	Leu129c	Lys148	
454	Tyr134	Lys148	HB
535	Phe204a	Arg143	HB
		Lys148	
		Gly149	

Appendix Table S9: F1 interactions with the SP in prothrombin. Contacting residues identified by the program 'contact' are listed. 8 HB and 5 SB identified by PISA. Chymotrypsin numbering used for SP domain of prothrombin.

F1	Prothrombin SP		
Residue	Residue	Chymo #ing	Interaction Type
Glu85	Arg498	173	2SB
Leu88	Asp552	221	
	Arg553	221a	
Arg90	Trp468	147a	
	Thr469	147b	
	Asp552	221	HB, 2SB
	Asp554	222	SB
Ser91	Trp468	147a	
	Trp547	215	
	Asp552	221	
Arg92	Tyr367	60a	
	Trp370	60d	
	Glu466	146	HB
	Thr467	147	HB
	Trp468	147a	
	Thr469	147b	
	Ala470	147c	
Tyr93	Tyr367	60a	
	Glu414	97a	
	Asn415	98	
	Leu416	99	HB
	Trp468	147a	
	Trp547	215	
Pro94	Tyr367	60a	HB
	Pro369	60c	

	Trp370	60d	
His95	Tyr367	60a	
	Pro369	60c	
	Trp370	60d	
Lys96	Pro369	60c	
	Trp370	60d	
Thr129	Glu414	97a	
Asp130	Arg413	97	
	Glu414	97a	
Pro131	Pro369	60c	
	Trp412	96	
	Arg413	97	
	Glu414	97a	
	Asn415	98	
Thr132	Trp412	96	
	Arg413	97	2HB
Arg134	Pro369	60c	HB
	Trp370	60d	

Appendix Table S10: F1 clashes (<2.3Å) with the SP in meizothrombin. Contacting residues identified by the program 'contact' are listed. 0 HB and 2 SB identified by PISA. Chymotrypsin numbering used for SP domain of meizothrombin.

F1	Meizothrombin SP		
Residue	Residue	Chymo #ing	distance (Å)
Glu85	Arg498	173	1.57
Arg92	Trp370	60d	1.31
Tyr93	Leu416	99	1.70
His95	Trp370	60d	1.52
Lys96	Trp370	60d	1.26
Pro131	Arg413	97	1.58
Arg134	Pro369	60c	1.83

Appendix Table S11: K2 contacts (<5Å) with the SP in prothrombin. Contacting residues identified by the program 'contact' are listed. 1,879.8 $Å^2$ buried; 3 HB and 15 SB identified by PISA. Chymotrypsin numbering used for SP domain.

K2	Prothrombin SP		
Residue	Residue	Chymo #ing	Interaction Type
Arg174	Arg418	101	
	Asp503	178	4SB
	Arg565	233	
Thr186	Pro408	92	
	Arg409	93	
	Asn411	95	
His187	Pro408	92	
	Tyr410	94	
	Asn411	95	
	Arg413	97	
His205	Gln571	239	
	Asp575	243	SB

Gln206	Gln571	239	
	Asp575	243	
Asp223	Lys568	236	
Gly224	Lys568	236	
Asp225	Arg565	233	
	Lys568	236	
	Trp569	237	
	Lys572	240	SB
Glu226	Arg443	126	
	Phe564	232	
	Arg565	233	SB
	Lys568	236	
Glu227	His407	91	
	Arg409	93	
	Arg418	101	4SB
	Asn504	179	HB
	Arg565	233	SB
Trp230	Lys572	240	
Lys236	Glu579	247	
Pro237	Gln576	244	
	Glu579	247	
Gly238	Gln576	244	
Asp239	Gln576	244	
Phe240	Pro408	92	
	Trp569	237	
	Lys572	240	
	Val573	241	
Gly241	Pro408	92	
Tyr242	His407	91	HB
	Pro408	92	
	Arg409	93	
	Trp569	237	
Asp244	Arg409	93	SB
Cys248	Asp503	178	
Glu249	Arg490	165	SB
	Lys494	169	SB
	Ile501	176	
	Thr502	177	
	Asp503	178	
Glu250	Asp503	178	HB
	Arg565	233	

Appendix Table S12: K2 contacts (<5Å) with the SP in meizothrombin. Contacting residues identified by the program ‘contact’ are listed. 1,579.1 Å^2 buried; 8 HB and 10 SB identified by PISA. Chymotrypsin numbering used for SP domain.

K2	Meizothrombin SP		
Residue	Residue	Chymo #ing	Interaction Type
Leu189	Arg413	97	
Leu202	Pro408	92	
Lys204	Lys572	240	HB
	Gln576	244	
His205	Trp569	237	

	Lys572	240	HB
	Gln576	244	
Gln206	His407	91	
	Pro408	92	
	Arg409	93	
	Trp569	237	
	Lys572	240	HB
Asp207	Lys568	236	
Asp223	Arg409	93	
	Arg418	101	
Asp225	Arg409	93	SB
	Asp417	100	
	Arg418	101	3SB
	Thr502	177	
Glu226	Arg490	165	
	Arg500	175	
	Ile501	176	
	Thr502	177	
	Asp503	178	HB
Glu227	Glu414	97a	
	Arg500	175	4SB
	Ile501	176	
	Thr502	177	
Trp230	Arg409	93	
Tyr232	Pro408	92	
	Arg409	93	
Pro237	Leu366	60	
	Ile406	90	
	His407	91	
	Pro408	92	
	Tyr410	94	
	Trp412	96	
Gly238	His407	91	
	Pro408	92	HB
	Tyr410	94	
	Asn411	95	
	Trp412	96	
	Arg413	97	HB
Asp239	Pro408	92	
	Trp412	96	
	Arg413	97	2SB
Phe240	Pro408	92	
	Arg409	93	
	Tyr410	94	
	Asn411	95	
	Arg413	97	
	Arg418	101	
Tyr242	Arg409	93	
	Asn411	95	HB
	Glu414	97a	HB
	Asp417	100	
	Arg500	175	

Appendix Table S13: K2 in prothrombin position clashes (<2.3Å) with the SP in meizothrombin. Contacting residues identified by the program 'contact' are listed. Chymotrypsin numbering used for SP domain of meizothrombin.

K2	Meizothrombin SP		
Residue	Residue	Chymo #ing	distance (Å)
Thr186	Arg409	93	1.37
Ser203	Gln576	244	2.23
Asp225	Lys568	236	2.17
Phe240	Trp569	237	2.20
Tyr242	His407	91	1.29
Glu249	Arg490	165	1.98
Glu250	Arg565	233	2.26

Appendix Table S14: List of primers to generate pET23-M17 (EGF2-SP) S195A construct.

Primer name	Sequence (5'-3')
pET23_M17_inv_reverse	ccccgttgaacgccctgcagagcttccgcattttaatttctcctctttaatgaattcggatcctagaggg
EGF2_M17_forward	cattaaagaggagaaattaaaatgcggaagctctgcagggcgttcaacggggactgtgaccagttctgca agagggtacagagctctgtggtgtgctcctgc
L1_M17_forward	ccgagcgtgactgggccaatgagacgctgatgaagcaggatacggggattgtgagcgg
L1_M17_reverse	ccgctcacaatccccgtatcctgcttcatcagcgtctcattggcccagtcacgctcgg
L2_M17_forward	ggaggtgccctacgtggaccgccacagctgcatgctgtccagcgacttcaggatcacccagaa
L2_M17_reverse	ttctgggtgatcctgaagtcgctggacagcatgcagctgtggcggtccacgt
S195A_forward	cagggggacgccgggggcccgcacgtcacccgcttcaaggacacctacttc
S195A_reverse	ggtgacgtgcgggcccccggcgtccccctggca
pET23_M17_inv_forward	atgaaaaccaggggcttgcccaaggccaagtaatgaagcttgcggccgcactcgag
L3_M17_reverse	ttacttggccttgggcaagcccctggttttcatggacctcttgatccacttgaggaagcgggtgaccttggt gtag

Appendix Table S15. Assembly of pET23- M17 (EGF2-SP) S195A construct using Gibson Assembly method as described by the Manufacturer's instructions. Vector backbone (fragment1) was amplified from pET23 (+) plasmid (lab stock) and M17 mutations were introduced using specific primers as described in Appendix Table S14. pCEP4-fX FL (Üstok and Huntington, 2026) plasmid was used as a templated to introduce M17 mutations and generate fragments shown below. These fragments were PCR purified, gel extracted and used in assembly procedure.

Fragment No	Primer Pair	Template DNA	Fragment Size (bp)
1	pET23_M17 inv_reverse<br>pET23_M17_inv_forward	pET23 (+)	3679
2	EGF2_M17_forward<br>L1_M17_reverse	pCEP4-fX FL	737
3	L1_M17_forward<br>L2_M17_reverse	pCEP4-fX FL	172
4	L2_M17_forward<br>S195A_reverse	pCEP4-fX FL	136
5	S195A_forward<br>L3_M17_reverse	pCEP4-fX FL	183
