# Supplementary material for: Prothrombinase processivity is conferred by substrate allostery
Source: EMBO J. 2026 Apr 22;45(11):3954–77. doi: 10.1038/s44318-026-00782-4 (PMC13226733; doi:10.1038/s44318-026-00782-4)
Supplement: Supplementary file 2 — Table EV1 [file 44318_2026_782_MOESM2_ESM.docx]

**Table EV1.** Cryo-EM data collection, refinement and validation statistics for the prothrombinase-prothrombin complex (EMD- 56052; PDBID 9TLE).

|  | | Prothrombinase-Prothrombin Complex | |
| --- | --- | --- | --- |
| **Sample preparation**  Buffer  Concentrations (μM)  Sample volume (μl)  Grid type  Glow discharge time (s)  Glow discharge current (mA)  Blotting chamber temp. (°C)  Blotting chamber humidity (%)  Blot time (s)  Blot force (N)  **Data collection and processing** | | | HEPES pH 7.5, NaCl, CaCl_2_  0.65 fVa, 3.9 fXa, 1.3 prothrombin  3  QuantiFoil (AU) R1/1 300 mesh  60  25  4  100  1  -7 |
| Voltage (kV) | 300 | | |
| Detector mode | Counting – LZW-TIFF output | | |
| Indicated magnification | 130,000× | | |
| Pixel size (Å) | 0.829 | | |
| C2 aperture (μm) | 50 | | |
| Defocus range (μm) | -1.8 to -0.6 | | |
| Zero-loss slit width (eV) | No energy filter | | |
| Exposure rate (e/pix/s)  Exposure rate (e/Å^2^/s)  Exposure time (s) | 12.63  18.38  2.72 | | |
| Total exposure (e-/Å^2^) | 50.0 | | |
| Movies collected | 24,030 | | |
| **Refinement & validation** |  | | |
| Symmetry imposed | C1 | | |
| Initial particle images (#) | 7,138,515 | | |
| Final particle images (#)  Map resolution (Å) | 76,039  3.11 | | |
| Model composition |  | | |
| Non-hydrogen atoms | 18,403 | | |
| Protein atoms | 17,912 | | |
| Ions  Carbohydrate atoms | 2  489 | | |
| R.m.s. deviations |  | | |
| Bond lengths (Å) | 0.004 | | |
| Bond angles (º) | 0.755 | | |
| Ramachandran favoured (%)  Ramachandran outliers (%)  MolProbity score  Clash score  Rotamer outliers (%) | 89.92  0.41  2.36  20.47  0 | | |
|  |  | | |
