# Supplementary material for: Prothrombinase processivity is conferred by substrate allostery
Source: EMBO J. 2026 Apr 22;45(11):3954–77. doi: 10.1038/s44318-026-00782-4 (PMC13226733; doi:10.1038/s44318-026-00782-4)
Supplement: Supplementary file 4 — Table EV3 [file 44318_2026_782_MOESM4_ESM.docx]

**Table EV3.** Data collection, refinement and validation statistics for PDBID 9T00.

Highest resolution shell is in parenthesis.

|  | Pre-2:a2-peptide | | |
| --- | --- | --- | --- |
| **Data collection** | |  | |
| Space group | | | P1 |
| Cell dimensions | | |  |
| *a*, *b*, *c* (Å) | | | 51.42, 51.57, 65.99 |
| α,β,γ (°) | | | 82.90, 85.33, 65.90 |
| Resolution (Å) | | | 38.76-2.80 (2.95-2.80) |
| *R_merge_* | | | 0.244 (1.03) |
| Mean *I*/σ*I* | | | 3.0 (0.7) |
| CC_1/2_  Completeness (%) | | | 0.950 (0.423)  99.2 (98.9) |
| Redundancy | | | 3.7 (3.4) |
| **Refinement & validation** | | |  |
| Resolution (Å) | | | 38.76-2.80 |
| No. reflections | | | 14,261 |
| *R*_work/_ *R*_free_  (2.87-2.80 Å) | | | 0.259/0.305  (0.414/0.392) |
| No. atoms | | |  |
| Protein/peptide | | | 5,114 |
| Water | | | 11 |
| B-factors | | |  |
| All | | | 48.0 |
| R.m.s. deviations | | |  |
| Bond lengths (Å) | | | 0.001 |
| Bond angles (º) | | | 0.785 |
| Ramachandran favoured (%)  Ramachandran outliers (%)  MolProbity score | | | 90.41  1.46  2.73 (82^nd^ percentile) |
|  | | |  |
|  | | |  |
