# Supplementary material for: Prothrombinase processivity is conferred by substrate allostery
Source: EMBO J. 2026 Apr 22;45(11):3954–77. doi: 10.1038/s44318-026-00782-4 (PMC13226733; doi:10.1038/s44318-026-00782-4)
Supplement: Supplementary file 7 — Movie EV2 [file 44318_2026_782_MOESM7_ESM.zip › Movie_EV2_legend.docx]

**Movie EV2: Top view of SP domain rotation in the transition from prothrombin to meizothrombin.** A morph of the serine protease (SP) domain (coloured blue-to-red from N-to-C terminus) of prothrombin to the SP position found in the meizothrombin structure. Prothrombinase (grey) does not alter conformation.
