# Supplementary material for: Prothrombinase processivity is conferred by substrate allostery
Source: EMBO J. 2026 Apr 22;45(11):3954–77. doi: 10.1038/s44318-026-00782-4 (PMC13226733; doi:10.1038/s44318-026-00782-4)
Supplement: Supplementary file 8 — Movie EV3 [file 44318_2026_782_MOESM8_ESM.zip › Movie_EV3_legend.docx]

**Movie EV3: K2 domain repositioning upon conversion from prothrombin to meizothrombin.** A morph of the serine protease domain (coloured blue-to-red from N-to-C terminus) and the K2 domain and activation loop (pink) illustrates changes during the transition from prothrombin meizothrombin.
