# Supplementary material for: Prothrombinase processivity is conferred by substrate allostery
Source: EMBO J. 2026 Apr 22;45(11):3954–77. doi: 10.1038/s44318-026-00782-4 (PMC13226733; doi:10.1038/s44318-026-00782-4)
Supplement: Supplementary file 9 — Movie EV4 [file 44318_2026_782_MOESM9_ESM.zip › Movie_EV4_legend.docx]

**Movie EV4: Presentation of Arg271 to the active site of fXa after cleavage of Arg320.** A morph of the serine protease domain (cyan) and the K2 domain and activation loop (green) of prothrombin to meizothrombin. The side chains of Arg320 (yellow) and Arg271 (magenta) shown as sticks. Disulfide bonds are shown and prothrombinase (grey) is taken from the structure with prothrombin.
