# Supplementary material for: Prothrombinase processivity is conferred by substrate allostery
Source: EMBO J. 2026 Apr 22;45(11):3954–77. doi: 10.1038/s44318-026-00782-4 (PMC13226733; doi:10.1038/s44318-026-00782-4)
Supplement: Supplementary file 10 — Expanded View Figures [file 44318_2026_782_MOESM10_ESM.pdf]

Expanded View Figures

Figure EV1. Schematics of prothrombin processing and sequence of the a2-loop.

(**A**) Schematics of prothrombin domain organization in open and closed states. The N-terminal gamma carboxyglutamic acid (Gla) domain and the first kringle domain (K1) are associated and are followed by a 26-residue linker and the K2 and serine protease (SP) domains that are also tightly associated. The closed form, where K1 interacts with the active site of the SP domain, predominates in solution. (**B**) Prothrombin processing pathways are shown with domains labeled as in (**A**). fXa, on its own will cleave first at Arg271 (left) to form prethrombin-2 (Pre-2) and F1.2, and prothrombinase cleaves initially at Arg320 to activate the SP domain (red) and form meizothrombin. The second cleavage event leads to thrombin and F1.2 as products. (**C**) The sequence of the a2-loop of factor fVa is given, with the two acidic regions containing sulfated tyrosines underlined. Residues are numbered, and * denotes sulphation.

© The Author(s)

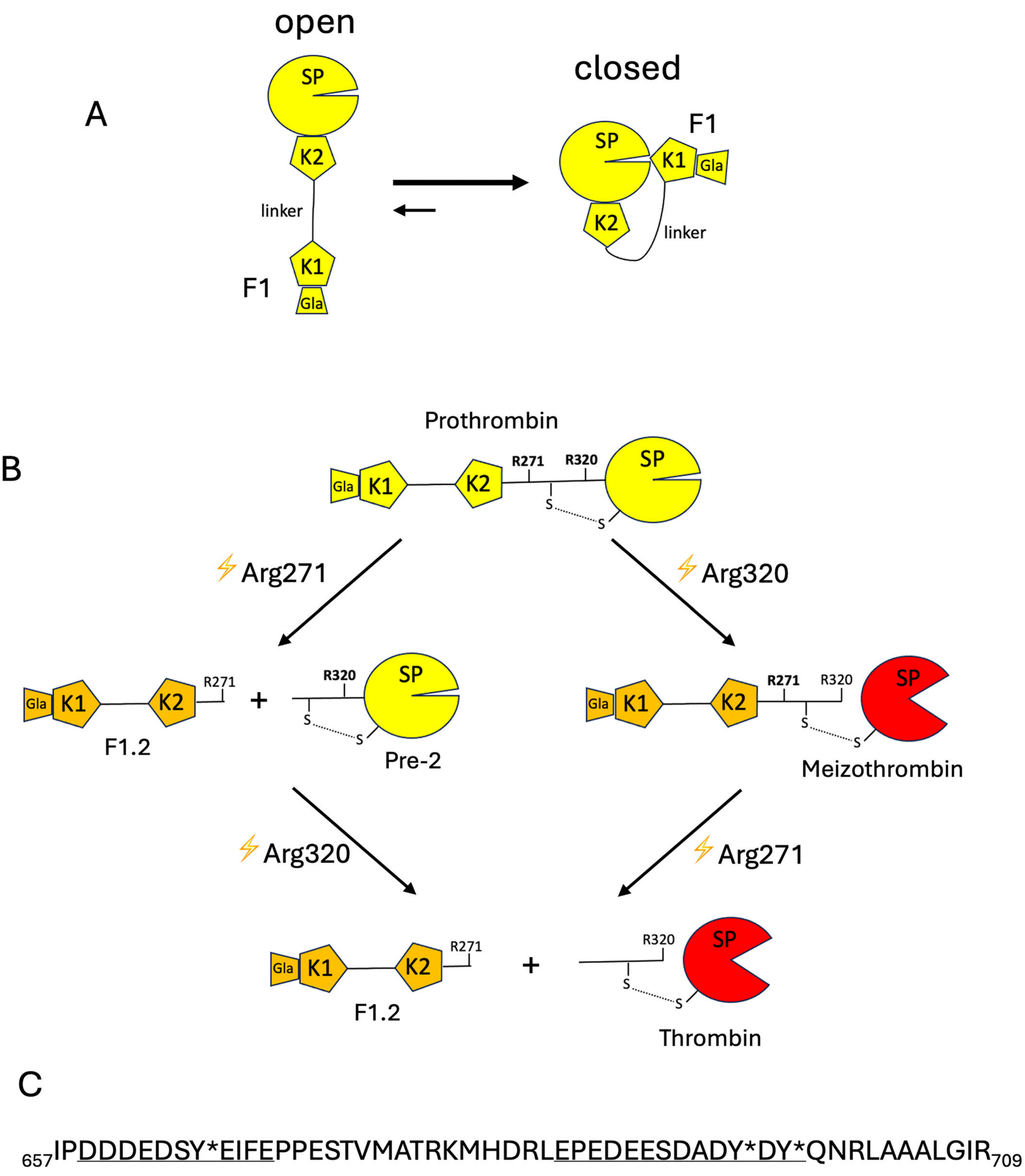
A
open
closed
SP
K2
linker
K1
F1
Gla
B
Prothrombin
R271
R320
S
Arg271
Arg320
F1.2
Pre-2
Meizothrombin
Thrombin
C
657IPDDDEDSY*EIFEPPESTVMATRKMHDRLEPEDEESDADY*DY*QNRLAAALGIR709

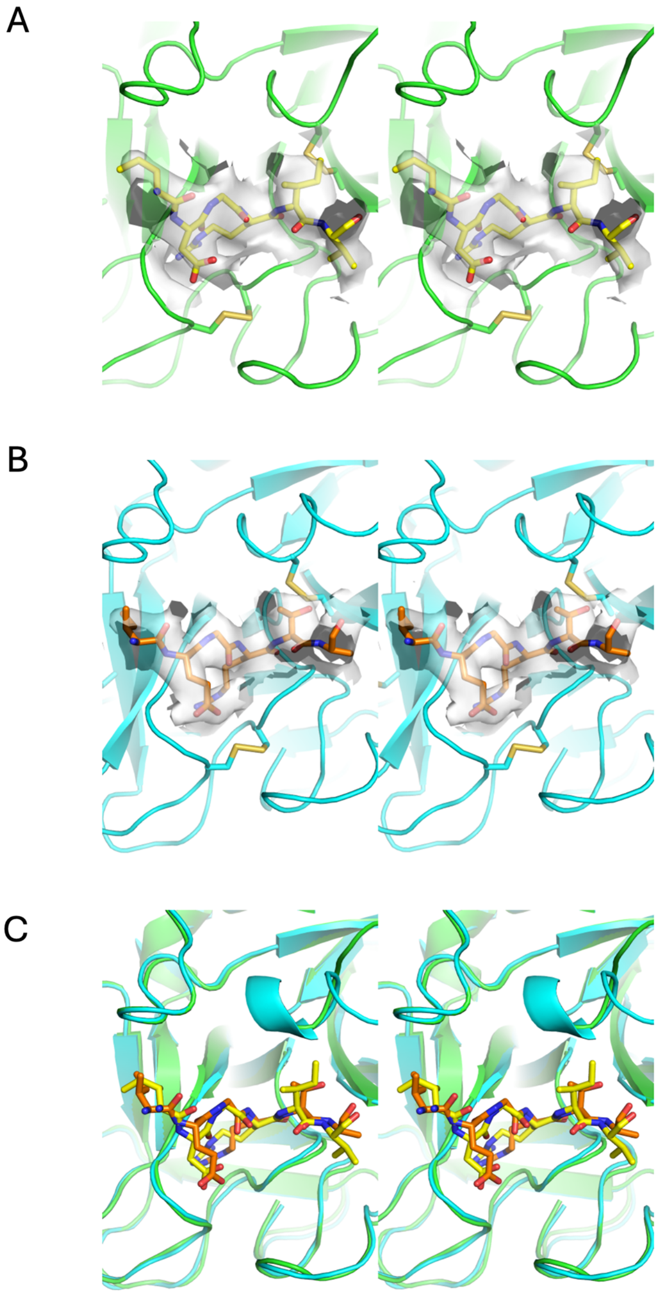

Figure EV2. Substrate loop interactions within the active site of fXa.

(**A**) Stereo view of the active site of fXa (green) from the prothrombinase-prothrombin complex, with residues 317-322 of prothrombin (yellow sticks) surrounded by map (semitransparent gray). (**B**) Stereo view of the active site of fXa (cyan) from the prothrombinase-meizothrombin complex, with residues 268-273 of meizothrombin (orange sticks) surrounded by map (semitransparent gray). (**C**) Superposition of the prothrombin and meizothrombin interactions in the active site of fXa, colored as in (**A**, **B**). The figures are in the standard orientation, so that the N-terminal portion of the substrate loop is on the left and the C-terminus on the right.

 © The Author(s)

A

B

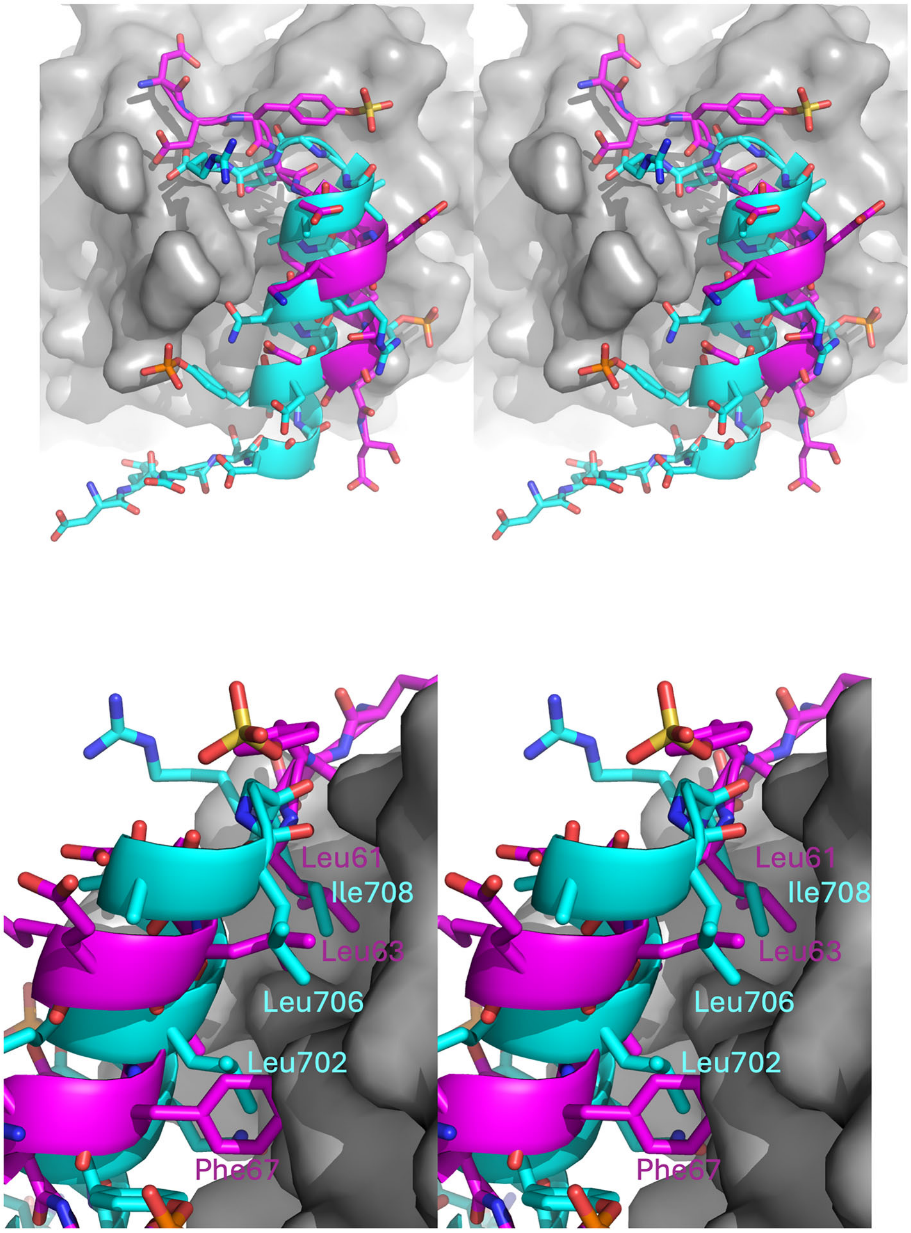

Figure EV3. Comparison between a2-loop C-terminal and HCII N-terminal interactions with exosite I.

(**A**) Stereo view of the a2-loop (cyan) interaction with prothrombin (gray surface) exosite I in the prothrombinase-prothrombin complex and the interaction of the N-terminal acidic region of heparin cofactor II (HCII; magenta) with exosite I of thrombin. (**B**) The helices run in opposite directions, but both place key hydrophobic side chains in remarkably similar positions: Phe67, Leu63, and Leu61 for HCII and Leu702, Leu706, and Ile708 for fVa.

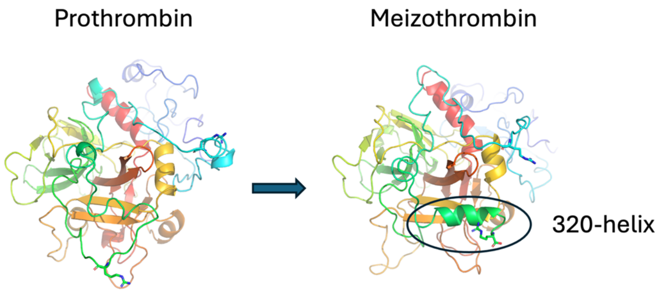

Figure EV4. Transition of the 320-loop of prothrombin into a helix after cleavage at Arg320 to produce meizothrombin.

The K2 and SP domains are shown as cartoons, colored from N-to-C-terminus (blue-to-red). The new 320-helix is indicated.

 © The Author(s)

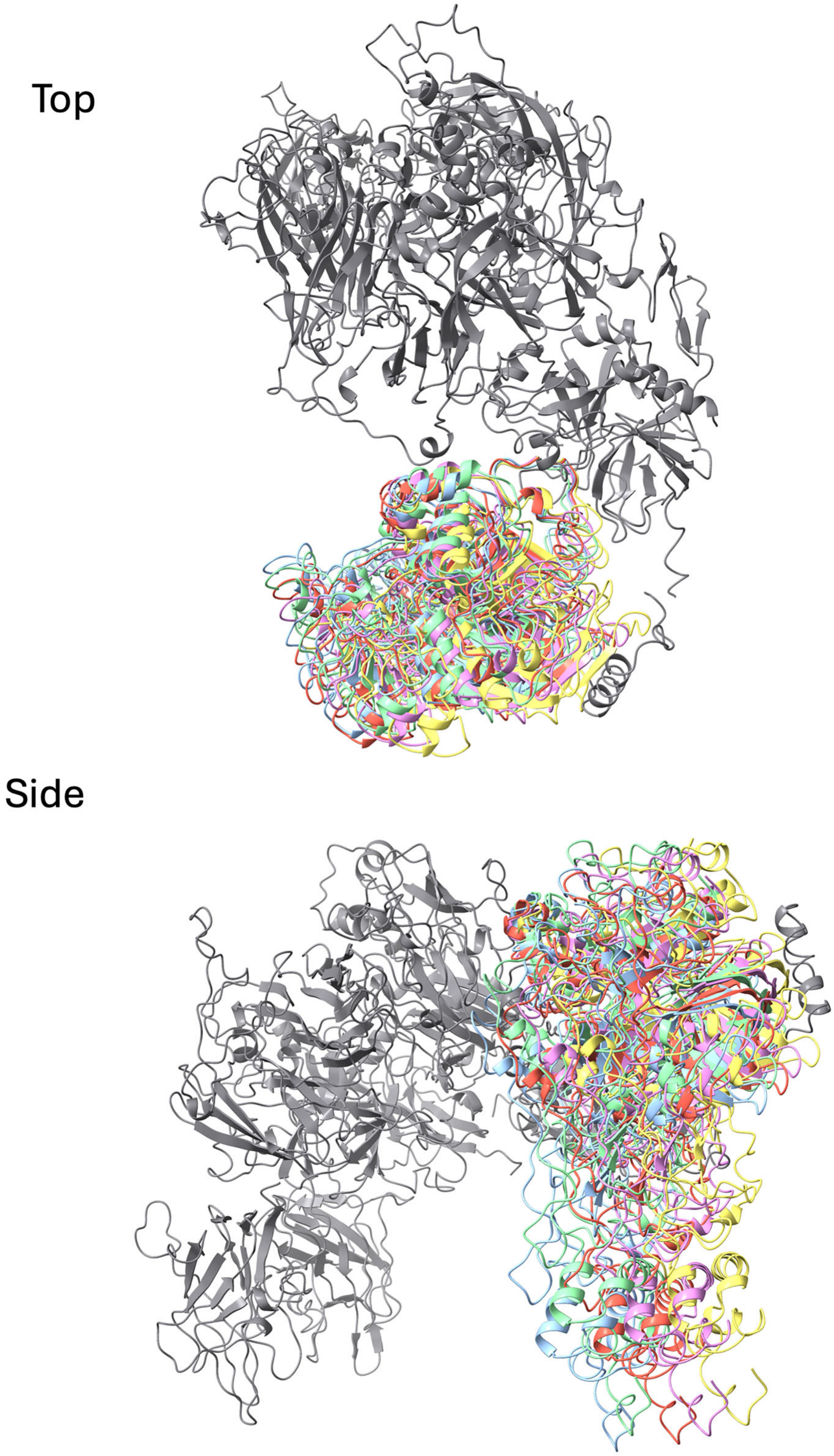

Figure EV5. Five particle subset classes of the prothrombin component in the prothrombinase complex is illustrated by the fit of prothrombin into the resulting maps.

Top and side views are shown. Gray is prothrombinase, and the colored cartoons are prothrombin.

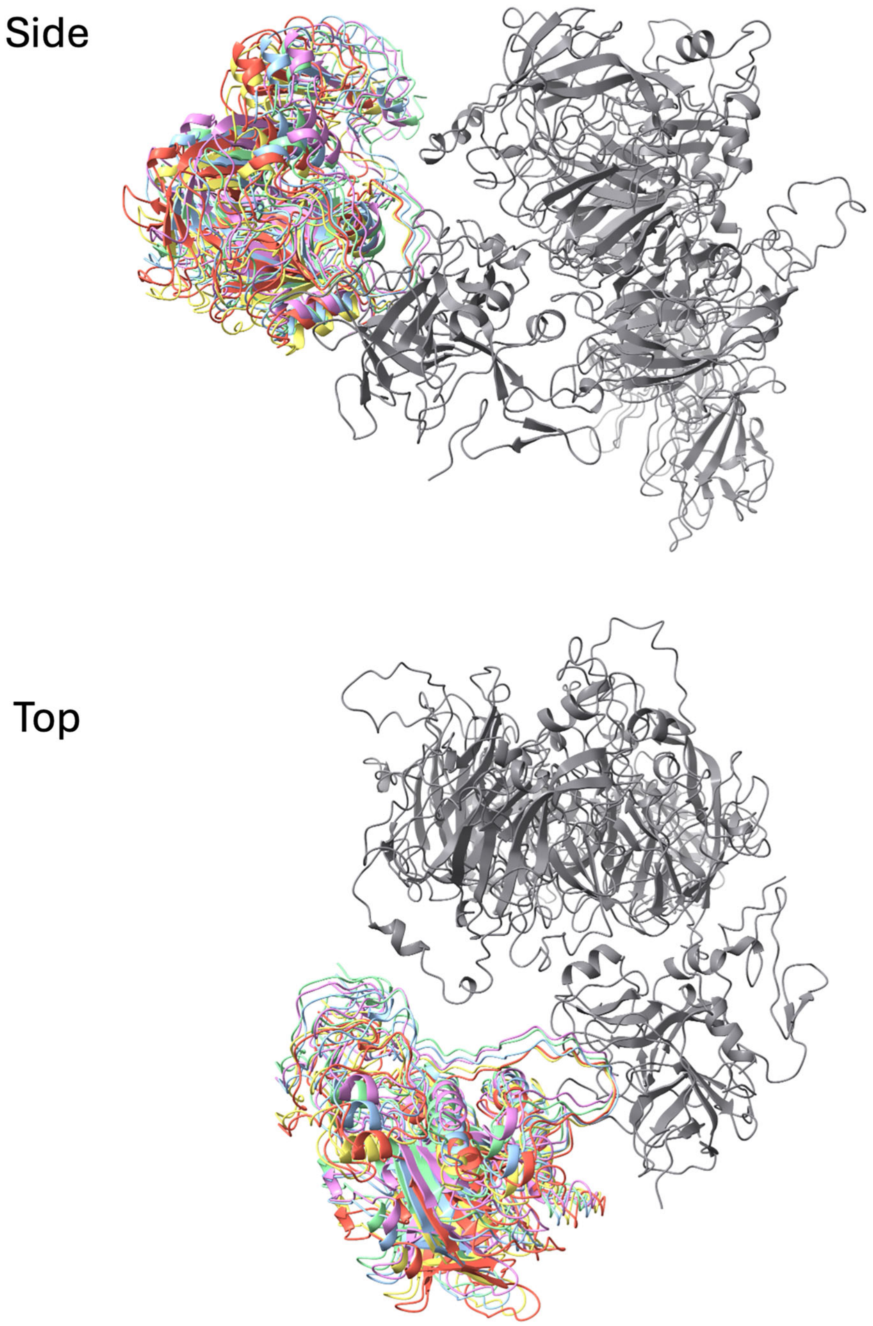

Figure EV6. Five particle subset classes of the meizothrombin component in the prothrombinase complex is illustrated by the fit of meizothrombin into the resulting maps.

Side and top views are shown. Gray is prothrombinase, and the colored cartoons are prothrombin.

 © The Author(s)
